# Are parental sociodemographic factors, oral health knowledge and practices linked to the oral health habits of their children with cleft lip and palate?

**DOI:** 10.2340/aos.v83.40938

**Published:** 2024-06-19

**Authors:** Dilan Altun, Sevilay Karahan, Fatma Figen Özgür, Meryem Uzamış Tekçiçek, Melek Dilek Turgut

**Affiliations:** a Department of Pediatric Dentistry, Faculty of Dentistry, Hacettepe University, Ankara, Turkey; b Department of Biostatistics, Faculty of Medicine, Hacettepe University, Ankara, Turkey; c Department of Plastic Reconstructive and Aesthetic Surgery, Faculty of Medicine, Hacettepe University, Ankara, Turkey

**Keywords:** Cleft lip and palate, children, oral health

## Abstract

**Objective:**

Children with cleft lip and palate (CLP) have a greater risk of dental caries. The parents’ knowledge and attitudes may have an impact on their children’s oral health and dietary habits. Therefore, the aim of this study was to assess the socio-demographic characteristics, oral health knowledge, oral health behaviours, and habits of the parents in addition to the relationship with the oral health and dietary practices of their children with CLP.

**Material and methods:**

The parents of 343 patients with CLP participated in the study. An online questionnaire with 52 questions regarding sociodemographic characteristics, oral health and oral hygiene practices was presented to them.

**Results:**

Parents with higher level of education had better oral health knowledge (*p* < 0.05). Logistic regression analysis showed that the factors affecting the child’s tooth brushing habits were the mother’s age (odds ratio [OR] = 1.071, 95% confidence interval [CI]: 1.062–1.153), the father’s employment status (OR = 2.089, 95%CI: 1.065–4.097), and the mother’s last dental visit (OR = 1.995, 95%CI: 1.119–3.557). The factors affecting the child’s toothpaste usage were the mother’s age (OR = 1.106, 95%CI: 1.030–1.114), the father’s employment status (OR = 2.124, 95%CI: 1.036–4.354), and the mother’s last dental visit (OR = 2.076, 95%CI: 1.137–3.79).

**Conclusions:**

Parental factors have a significant influence on the oral health-related behaviours of children with CLP.

## Introduction

Cleft lip and palate (CLP), which may include the lip, alveolus, and palate in various combinations, is one of the most common congenital anomalies in the head and neck region. The general prevalence of CLP is approximately 1 in 700 live births [1].

Children with CLP frequently have complex medical and dental conditions including hearing difficulty, speech and language disorders, middle ear abnormalities, psychosocial problems, and dental anomalies. These conditions could have a long-term negative impact on a child’s health and ability to integrate into society, and they require interdisciplinary care from infancy through adulthood [2]. Treatment strategies for children with CLP aim at improving both function and aesthetics. However, the programmes often focussed on surgical care and place less emphasis on general oral health care. Yet, the outcome or success of treatment also depends on good dental health [3].

Although some studies reported no increased caries risk in CLP patients [4, 5], according to a recent systematic review and meta-analysis, individuals with CLP have a higher caries prevalence than the healthy population, both in deciduous and permanent dentition [6]. It is more challenging to provide optimal oral and dental care to children with CLP because of the complex anatomy of the cleft region and possible dental anomalies like supernumerary teeth, hypoplastic enamel defects and position anomalies, among others. [7]. In addition, dental caries appears to be closely related to oral health literacy. Assessment of the oral health knowledge and oral hygiene practices of parents may give insight into the oral health habits of their children [8].

There have been studies assessing the knowledge and habits of parents of children with CLP on oral and dental health [3, 8–10]. Nevertheless, it is worth noting that a limited number of patients were examined and only a narrow range of parameters were assessed in many of these studies. Therefore, the aim of this study was to evaluate the relationship between socioeconomic characteristics, oral health knowledge, oral health behaviours and habits of the Turkish parents of CLP children regarding oral health and dietary practices.

## Materials and methods

### Study design

The present cross-sectional study design was approved by the Non-Interventional Clinical Research Ethics Committee at Hacettepe University (approval no: 2020/17-02, date: 01.09.2020).

### Participants

The study targeted the parents of 731 patients with CLP aged 0–18 years who had been on regular follow-up by Hacettepe University Faculty of Medicine, Department of Plastic Reconstructive and Esthetic Surgery.

### Data collection

A self-administered anonymous online questionnaire with 52 questions was developed by the research team based on expert group discussions and literature review. In the initial phase of pre-testing for content and clarity assessment, the phrase ‘cleft lip and palate’ was excluded from the questionnaire and the initial draft of the questionnaire was administered to 10 parents of children. The questions were reviewed, and the appropriate modifications were made in accordance with the feedback received. The first part of the questionnaire contained 22 questions about the sociodemographic characteristics, oral health status and oral hygiene practices of the parents (i.e., oral health, tooth brushing habits, dental visits, smoking habits). The second part contained 29 questions referring to the child’s CLP-related medical history (6 questions), birth characteristics (3 questions), eating habits (9 questions), oral health practices (9 questions), and speech problems (2 questions). By the final question, parents’ knowledge on oral and dental health practices for their children was assessed (i.e., reducing sugar consumption, regular dental check-ups, effective and regular tooth brushing, dental floss usage, treatment of decayed teeth, fluoride toothpaste and varnishes).

The study was conducted between October 2020 and February 2021, during the coronavirus disease 2019 (COVID-19) pandemic. Therefore, electronic questionnaires were sent to the parents of 731 eligible children with cleft lip and/or palate through text messages containing the electronic link. Reminders were sent out four additional times every 4 weeks after the initial text message. The data-collection period ended 5 months after the first text message was sent.

### Data analysis

The data were analysed using IBM Statistical Package for the Social Sciences (SPSS) Statistics 23.0 (IBM Corp., Armonk, NY, USA). Descriptive statistics included number, percentage, mean and standard deviation (SD), median and the smallest and largest values. The chi-square test, Fisher’s exact test and Mann-Whitney U test were utilised to evaluate whether the differences between categorical variables were statistically significant. Logistic regression analysis was performed to examine the association between parental factors (i.e., mother’s age, father’s occupational status, and the timing of the mother’s last dental visit) and the child’s oral health behaviours (i.e., the child’s toothpaste usage and tooth brushing habits). The statistical significance level of all analyses was accepted as *p* < 0.05.

## Results

Of the 731 parents who were sent the link, 343 responded by filling out the electronic questionnaire. The response rate thus was 46.9%.

### Descriptive characteristics of the children

As the age range of the children was 0–18 years, the participants were categorised according to paediatric age-stage terminology [11] – 167 (48.7%) were infants-toddlers (0–2 years), 133 (38.8%) were preschoolers (2–6 years), 24 (7.0%) were in school-age (6–12 years), and 19 (5.5%) were adolescents (older than 12 years). The mean age of the children was 3.28 (minimum 00.00, maximum 17.91) years. Boys outnumbered girls, with 199 (58%) of the children being boys and 144 (42%) being girls (*p* = 0.003). The distribution of the children by CLP type and gender is shown in Table 1. The association between the child’s gender and CLP type is statistically significant (*p* = 0.010). Regarding birth characteristics, 73.2% of the children were born at 38 weeks, and 60.9% of them were delivered by caesarean section. The average birth weight was 3.147 grams.

**Table 1:** Sample distribution according to cleft type and gender.

Cleft type	Boys		Girls		Total		*P*
	*n*	%	*n*	%	*n*	%	
Bilateral CLP	46	23.1	21	14.6	67	19.5	0.010*
Unilateral CLP	69	34.7	46	31.9	115	33.5	
Combined soft and hard cleft palate	23	11.6	14	9.7	37	10.8	
Isolated soft cleft palate	22	11.1	38	26.4	60	17.5	
Isolated cleft lip	17	8.5	12	8.3	29	8.5	
Unknown	22	11.1	13	9	35	10.2	

Chi-square test. CLP: cleft lip and palate.

* *p* < 0.05.

Among the children, 19.1% had a family member who also had CLP, 21.3% had another medical condition accompanying CLP, and 9.3% had been using regular medication. After birth, 37.0% of them used a nasoalveolar molding (NAM) appliance.

### Oral health habits of the children

According to the parents, 58.0% of the children had their own toothbrush. Regarding toothbrushing habits of the children, 13.7% brushed 2 or more times/day, 20.7% sometimes, and 19.2% once a day. About half of the parents (46.1%) reported that their children never brushed their teeth. Neither the child’s gender nor the CLP type showed any significant association with the toothbrushing frequency or toothpaste usage. Parents reported that 36.4% of their children visited the dentist less than a year ago and 14.3% more than a year ago, while 49.2% of the children had never visited a dentist or their parents couldn’t recall the last visit.

Table 2 demonstrates children’s last dental visit and their oral hygiene habits according to their age groups. Children’s age influenced their oral hygiene habits and the timing of their last dental visit (*p* < 0.05). According to the parents, all the children older than 6 years were brushing their teeth and using toothpaste. Overall, the children older than 6 years had higher rates of toothbrushing, toothpaste usage, and visited the dentist more recently.

**Table 2:** Children’s oral hygiene habits and timing of their last dental visit according to their age.

Age group	Toothbrushing (%)					Toothpaste usage (%)					Last dental visit (%)						
	Yes		No		*P*	Yes		No		*P*	<12 months		>12 months		Never/ Unknown		*P*
	*n*	%	*n*	%		*n*	%	*n*	%		*n*	%	*n*	%	*n*	%	
0–2 years	27	16.2	140	83.8	0.00*	22	13.2	145	86.8	0.00*	37	22.2	7	4.2	123	73.7	0.00*
2–6 years	113	85	20	15		91	68.4	42	31.6		57	42.9	31	23.3	45	33.8	
6–12 years	24	100	0	0		24	100	0	0		17	70.8	6	25	1	4.2	
>12 years	19	100	0	0		19	100	0	0		14	73.7	5	26.3	0	0	

Chi-square test.

* *p* < 0.05.

### Parental characteristics and effects on the oral health habits of the children

With respect to the obtained results, the mean age of the fathers (35.48) was higher than that of the mothers (31.38) (*p* = 0.00). The mean age of the parents of the children who brush their teeth or use toothpaste was significantly higher than that of the parents of the children who don’t brush their teeth or use toothpaste (*p* = 0.00 and *p* = 0.00 for toothbrushing and toothpaste usage, respectively). The employment rate was 18.4% and 86.3% for the mothers and fathers, respectively. While the rate of toothbrushing and toothpaste usage was significantly higher for children whose fathers were employed (*p* = 0.004 and *p* = 0.008, respectively for toothbrushing and toothpaste usage), their mothers’ employment status did not make any significant difference (*p* = 0.344 and *p* = 0.706 respectively, for toothbrushing and toothpaste usage).

In regard to the educational status, 65.3% of the mothers and 73.3% of the fathers were at least high school graduates. The fathers had a higher educational level than the mothers (*p* = 0.046). While the increase in the father’s education level and the child’s toothbrushing status was statistically significant (*p* = 0.027), there was no significant correlation between the mother’s education level and the child’s toothbrushing status (*p* = 0.214). Neither the mother’s nor the father’s educational background showed any significant association with the toothpaste usage of the children (*p* > 0.05).

The distribution of the parental knowledge on oral health-related practices of their children is presented in Table 3. There was a statistically significant difference between the responses and the educational status of the parents (*p* < 0.05). Nevertheless, there was no statistically significant difference between the mother’s education level and fluoride toothpaste and varnish usage. Overall, parents with higher education levels had more oral health knowledge.

**Table 3:** Parental choices for oral and dental health-related practices that are beneficial for their children according to parental education level.

Oral and dental health related practices	Mother’s education level *n* (%) (*n* = 343)									Father’s education level *n* (%) (*n* = 343)								
	Primary school or below		Middle school		High school		College/under-graduate or above		*P*	Primary school or below		Middle school		High school		College/under-graduate or above		*P*
	*n*	%	*n*	%	*n*	%	*n*	%		*n*	%	*n*	%	*n*	%	*n*	%	
Reducing sugar consumption	23	45.1	47	69.1	79	73.8	94	80.3	0.000*	16	43.2	40	72.7	83	74.1	104	74.8	0.002*
Regular dental check-ups	31	60.8	44	64.7	68	63.6	95	81.2	0.001*	20	54.1	33	60	79	70.5	106	76.3	0.023*
Effective and regular tooth brushing	33	64.7	51	75	82	76.6	105	89.7	0.001*	21	56.8	43	78.2	92	82.1	115	82.7	0.005*
Dental floss usage	11	21.6	11	16.2	34	31.8	45	38.5	0.007*	2	5.4	14	25.5	35	31.3	50	36	0.003*
Treatment of decayed teeth	22	43.1	38	55.9	68	63.6	85	72.6	0.002*	13	35.1	31	56.4	72	64.3	97	69.8	0.001*
Fluoride toothpaste and varnishes	7	13.7	5	7.4	16	15	25	21.4	0.083	0	0	6	10.9	22	19.6	25	18	0.020*
I don’t know	14	27.5	7	10.3	12	11.2	8	6.8	0.002*	11	29.7	8	14.5	11	9.8	11	7.9	0.003*

Chi-square test.

* *p* < 0.05.

Among the parents, 35.0% of the mothers and 29.4% of the fathers brushed their teeth twice or more a day. Most of the mothers (99.7%) and the fathers (97.4%) used toothpaste. No statistically significant relationship was found between the parental tooth brushing habits and tooth brushing or toothpaste usage of the children (*p* > 0.05). In terms of smoking habits, 82.2% of the mothers and 47.2% of the fathers were nonsmokers and no significant association was found between parental smoking habits and tooth brushing or toothpaste usage of the children (*p* > 0.05). While 23.4% of the mothers had never visited the dentist or couldn’t recall the last visit, this rate was 28.3% for the fathers.

Children’s last dental visit, their oral hygiene habits and parent’s last dental visit are presented in Table 4. The timing of the most recent parental dental visit had an impact on the oral hygiene habits of their children (*p* < 0.05). Overall, the children whose parents visited the dentist in the last 12 months were more likely to brush their teeth, use toothpaste and visit the dentist recently.

**Table 4:** Children’s last dental visit and oral hygiene habits according to parent’s last dental visit.

Children’s last dental visit and oral hygiene habits		Mother’s last dental visit *n* (%)							Father’s last dental visit *n* (%)						
		<12 Months		>12 Months		Never/Unknown		*P*	<12 Months		>12 Months		Never/Unknown		*P*
		*n*	%	*n*	%	*n*	%		*n*	%	*n*	%	*n*	%	
Child’ last dental visit	<12 Months	65	43.5	37	32.2	23	28.8	0.000*	63	46.7	34	30.9	28	28.6	0.000*
	>12 Months	18	12.2	23	20	8	10		13	9.6	23	20.9	13	13.3	
	Never / Unknown	65	43.9	55	47.8	49	61.3		59	43.7	53	48.2	57	58.2	
Child’s toothbrushing		87	58.8	66	57.4	30	37.5	0.005*	83	61.5	57	51.8	43	43.9	0.027*
Child’s toothpaste usage		79	53.4	52	45.2	25	31.3	0.006*	74	54.8	47	42.7	35	35.7	0.012*

Chi-square test.

* *p* < 0.005.

Table 5 demonstrates the effects of the parental factors on the children’s tooth brushing and toothpaste usage by logistic regression analysis. The analysis revealed that the children with older mothers were 1.106 (95%CI:1.062–1.153) times more likely to brush their teeth and 1.071 (95%CI: 1.030–1.114) times more likely to use toothpaste. The children whose fathers were employed were 2.124 (95%CI: 1.036–4.354) times more likely to use toothpaste and were 2.089 (95%CI: 1.065–4.097) times more likely to brush their teeth. Furthermore, in the case in which their mothers visited the dentist within the previous year, the children were 2.076 (95%CI: 1.137–3.791) times more likely to use toothpaste and 1.995 (95%CI: 1.119–3.557) times more likely to brush their teeth.

**Table 5:** Logistic regression derived coefficients (OR), 95% confidence intervals, and P values for the association between parental factors and oral health habits of children.

Parental factors	Toothbrushing (yes/no)			Toothpaste Usage (yes/no)		
	*P*	OR	95% CI	*P*	OR	95% CI
Mother’s age	0.001	1.071	1.030–1.114	0.000	1.106	1.062–1.153
Father’s employment status (yes/no)	0.032	2.089	1.065–4.097	0.040	2.124	1.036–4.354
The timing of the mother’s last dental visit	0.061			0.037		
The timing of the mother’s last dental visit (in past 12 months /never)	0.019	1.995	1.119–3.557	0.017	2.076	1.137–3.791
The timing of the mother’s last dental visit (prior to past 12 months /never)	0.080	1.725	0.937–3.177	0.408	1.308	0.693–2.471

Logistic regression analysis.

CI: confidence interval; OR: odds ratio.

### Feeding practices

**Figure 1:**
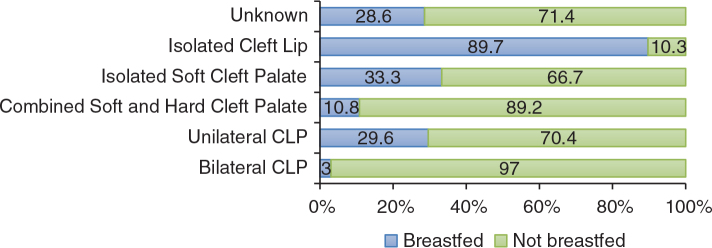
Sample distribution according to cleft type and breastfeeding practice.

Approximately three-quarters of the mothers (71.4%) reported that they had never breastfed their children. Most of the children (96.2%) used a feeding bottle for a while and 82.2% of the parents reported that they had bottle-fed their children at night. Formula (83.1%) and breast milk (64.7%) were the two most popular nutrients that these parents gave to their children in bottles at night. There was no significant correlation between the parental education level and the bottle-feeding practice at night (*p* > 0.05). The distribution of breastfeeding practice and CLP type are presented in Figure 1. According to the results of the chi-square test, breastfeeding was affected by the CLP type of the children (*p* = 0.00). There was no statistically significant relationship between the parental education level and breastfeeding or bottle-feeding practice (*p* > 0.05). Overall, breastfeeding prevalence in children with cleft involving the palate (i.e., bilateral CLP, combined cleft palate, unilateral CLP, etc.) was significantly lower (*p* < 0.05).

Considering the children’s beverage consumption, a small percentage (5.8%) of the parents reported that their children had been consuming sugar-sweetened beverages on a daily basis. The consumption of sugar-sweetened beverages in children of less educated parents was statistically higher than that of the children of highly educated parents (*p* < 0.05).

The most common practices leading to oral bacterial transmission from the parents to the children were sharing a spoon when feeding the child (38.2%), followed by kissing the child on the lips (10.2%) and cleaning the child’s pacifier in the parent’s mouth (7.9%). While the parental education level did not show a significant association with these practices, the avoidance of these practices increased significantly as the education level of the mother increased (*p* = 0.006).

## Discussion

This is the first study carried out in Turkey to assess the relationship between socioeconomic characteristics, oral health behaviours and habits of the parents with children with CLP towards their children’s oral health and nutritional habits.

Cleft lip and palate is more common in boys than girls, and unilateral CLP is more common than bilateral CLP [12–15]. Consistently, boys outnumbered girls significantly, and unilateral CLP was significantly higher than bilateral CLP in the present study.

Our results showed that fathers outnumbered mothers in both education level and being employed (*p* < 0.05). Likewise, in the recent Turkey Family Structure Survey 2021 provided by the Turkish Statistical Institute, it was reported that 15.4% of women had higher education levels than their husbands, whereas 39.8% of women were married to men with higher educational levels. The employment rate for men was 59.8% and for women it was 26.3% [16]. Regardless of their education or employment status, parents should perform toothbrushing for children after the eruption of the first primary tooth [17]. Jahandideh et al. [18] reported that Turkish parents did not have enough knowledge about the age of starting toothbrushing. The significantly lower toothbrushing rates for younger children in the present study also confirm this finding for the parents of the children with CLP. It could also be hypothesised that the presence of babies with unerupted primary teeth may have contributed to the reduced toothbrushing rates in the youngest age group.

With increasing age, children have developed the fine motor skills required for brushing their teeth [19]. Parental supervision is especially recommended until the child is at least 7 years of age [20]. Thornton-Evans et al. [21] reported that the toothbrushing frequency of children was significantly higher in older age groups in the national Health and Nutrition Examination Survey of the United States in 2013–2016. In Turkey, the recent nationwide epidemiologic study conducted in 2018 showed the rate of children who brush their teeth twice or more times a day by age 5 as 20.2%, by age 12 as 21.0%, and by age 15 as 28.2% [22]. In children with CLP, Zhu et al. [23] reported no significant difference in toothbrushing frequency among children with CLP in the 3–5, 6–12, and 13–21 age groups in China. In our study, all of the children aged 6 and above were brushing their teeth. This result could be attributed to the improved self-awareness of the children and their increased fine motor development with age. As these children are also students, school may also play a significant role in instilling the practise of tooth-brushing as part of teaching oral health care responsibilities.

Parental age is a significant socio-demographic factor that has a direct impact on their children’s dental health [24] and increasing maternal age is associated with better oral health habits and a lower prevalence of caries in the child [25, 26]. The present study showed that as the parental age increased, the rate of children’s toothbrushing and toothpaste usage increased, and logistic regression analysis results indicated that children with older mothers were more likely to brush their teeth and use toothpaste. It would be reasonable to attribute these to the parents’ advancing knowledge and experience with age.

As for the other socioeconomic status indicators, family income and parental employment status are also associated with the oral health status of children [27]. The higher prevalence of dental caries in children is associated with lower social status [28, 29] and lower family income [30, 31]. In the present study, logistic regression analysis showed that the children whose fathers were employed were more likely to use toothpaste and brush their teeth. In our society, it is a common tradition for mothers to take on the primary role of caring for the child while fathers typically assume the responsibility of working and providing for the household. Fathers who are employed can more readily afford to provide their children with toothbrushes and toothpaste, this fact therefore might have had an impact on the findings of our study.

Parental knowledge of oral and dental health increases along with their educational level and this influences how their children acquire positive oral health behaviours [32–35]. Chen et al. [34] reported that oral health literacy of the parents and positive oral health behaviours in children increased along with the educational level of the parents. Furthermore, they suggested that the mother’s educational level has a bigger influence on the oral health behaviours of the family. In contrast, it was found in the present study that the child’s toothbrushing rate increased along with the father’s education level, while there was no correlation with the mother’s education level. This difference could be attributed to the fact that the participants in our study were the parents of the children with CLP. Furthermore, differences in the social and family structures of the two countries might have played a role. Consistent with the findings of Chen et al. [34], our results showed that oral health knowledge level increases with the increase in parental education level. Nevertheless, some mothers were in doubt about fluoride toothpaste and varnishes regardless of their educational background. The mothers are often in charge of child-care in our society. Therefore, it would be reasonable to conclude that mothers with a lower educational background could be more influenced by the unreliable myths about fluoride in the social media. The rate of reporting fluoride toothpaste and varnishes as beneficial increased with the increase in the father’s education level, but the level of reporting was still quite low compared to other responses. These outcomes demonstrate that the society requires comprehensive and effective education and/or information about the benefits of topical fluorides.

According to Poutanen et al. [36], parental behaviours were more importantly connected with children’s behaviours than parental knowledge and attitudes, supporting the idea that children learn behaviours from their parents. In parallel to this suggestion, our results demonstrated that the children whose parents visited the dentist in the last 12 months were more likely to brush their teeth, use toothpaste and visit the dentist more readily. It would also be reasonable to think that the parent’s visit to the dentist can affect the parental oral health knowledge and thereby behaviour of their children.

Since parents who smoke often lead unhealthy lifestyles and have limited awareness of oral health, they may exhibit reduced concern for both their children’s oral health and overall well-being [37, 38]. Laitala et al. [39] showed a weak correlation between maternal smoking habits and their behaviour towards their children, while Petrauskine et al. [40] reported no association between maternal smoking and mothers’ behaviour towards their children. Similar to the findings of these studies, there was no significant association between parental smoking and toothbrushing or toothpaste usage of the children in the present study as well.

A Chinese study conducted on 104 3-to 6-year-old children with CLP showed that 65% of the children had been bottle-fed and the prevalence of dental caries was higher in bottle-fed children [3]. It also reported that the bottle-feeding rate of the children increased along with the mother’s education level. In the present study, approximately two-thirds of the children were not breastfed at all and almost all of them had been bottle-fed for a time. There was no correlation between the parental education level and the breastfeeding or bottle-feeding practice. These findings might be explained by the fact that the children needed bottle-feeding, even if they were breastfed by their mothers. Babies with CLP have difficulty creating suction during feeding because the oral cavity is not adequately separated from the nasal cavity. As a result of this, the breastfeeding rate in children with CLP types that include cleft palate was significantly lower in the present study.

The importance of a multidisciplinary team approach for patients with CLP is indisputable. In Hacettepe University, each member of the multidisciplinary team plays a pivotal role in the overall care of patients with CLP. At specified times, the patients who were referred to or had been on regular follow-up by the department of plastic surgery have been evaluated by the multidisciplinary team. Those who needed further dental evaluation have been consulted with the department of paediatric dentistry, located in a separate building. In the present study, it was found that 49.2% of the children had never visited the dentist, or their parents couldn’t remember the last visit. Of those children, 73.0% were 0–2 years old and might not have needed further dental evaluation because of unerupted primary teeth. It is also reasonable to speculate that the parents might have hesitated to visit the dentist as the study had been carried out during the pandemic. Parental underestimation of the importance of regular dental visits might have been another factor.

In the current study, there have been a few limitations. As previously stated, the study was conducted during the COVID-19 pandemic which resulted in an enormously decreased referral of patients to dental clinics. Therefore, the data collected were based on parents’ self-reported behaviour and awareness through an online questionnaire, and this data could not be compared with the clinical examination findings of both the children and the parents. Even though self-reported outcome measures might be susceptible to socially desirable answers, the acceptable response rate in terms of expressing a specific population with CLP in this survey increases the validity of the findings. Moreover, the results of the survey confirm the findings of the previous studies that caries-preventive behaviour in children with CLP is positively related to the higher socioeconomic status of the family and the education level of the parents [3,8,10,15]. Another limitation of the study was the heterogeneity of the study group by age, since almost half of the participants (48.7%) were 0–2 years old. To clarify the effect of age on some factors, the participants were categorised according to paediatric age-stage terminology.

Despite the abovementioned limitations, this study nevertheless has certain advantages. To the best of our knowledge, this is the first study in Turkey to evaluate the relationship between socioeconomic characteristics, oral health behaviours, and the habits of parents with children with CLP towards their children’s oral health and nutritional habits. The outcomes of this study may offer useful options for clinicians to counsel parents as parental factors seem to have a significant influence on the oral health-related behaviours of children with CLP. In conclusion, informing parents from various socioeconomic backgrounds about the benefits of proper oral care would help increase their oral health literacy and awareness and hinder unnecessary concerns about oral health care, such as the detrimental effects of topical fluorides. Moreover, health care professionals could educate parents about avoiding oral bacterial transmission between caregiver and child, the importance of first and regular dental visits, eating habits, and their relationship to oral health and oral hygiene practices. Further studies are recommended to validate the results of our study by adjusting for the factors mentioned above.

## Conclusion

Within the limitations of this study, it can be concluded that parental factors may have an influence on the oral health-related behaviours of children with CLP. Determining parental knowledge and awareness on oral hygiene methods, nutrition, and regular follow-up, and then giving necessary education would improve not only theirs but also their children’s oral and dental health.
